# Students’ Learning Characteristics, Perceptions of Small-Group University Teaching, and Understanding Through a “Meeting of Minds”

**DOI:** 10.3389/fpsyg.2019.00444

**Published:** 2019-03-22

**Authors:** Evangelia Karagiannopoulou, Noel Entwistle

**Affiliations:** ^1^ University of Ioannina, Ioannina, Greece; ^2^ University of Edinburgh, Edinburgh, United Kingdom

**Keywords:** perceptions of teaching, learning intentions, learning characteristics, conceptions of knowledge, levels of understanding, small-group teaching, “meeting of minds”

## Abstract

Previous research has described some of the main characteristics of university teachers who teach in different ways, using a variety of methods and conceptions. What is generally missing from previous research is the impact of contrasting teaching approaches on students with different learning characteristics. The present investigation builds on a previous case study that identified the potential influence of a “meeting of minds” between tutors and students in developing personal understanding and also suggested contrasting perceptions of differing forms of teaching. Twenty-one in-depth interviews were used to identify distinctive perceived ways of teaching and groups of students with contrasting learning intentions, looking in particular at the perceptions of tutors who were seen to encourage a “meeting of minds.” The main characteristics of these tutors were found be *tolerating ambiguity, showing authenticity and empathy,* which led to *providing opportunities for discussion* in breaks*, and offering thinking spaces* within class. The analyses identified contrasting perceptions of teaching among students who differed in their learning characteristics and suggested how cognitive and affective elements in students’ experiences of teaching might be intertwined in influencing the development of personal understanding.

## Previous Research into Student Learning and University Teaching

Given space limitations, it has proved impossible to provide detailed reviews of the extensive research previously carried out on university teaching and student learning, but examples have been chosen to indicate the scope of the theoretical framework within which this study was conducted.

### University Teaching

Previous research on teaching explored students’ preferences for different types of teachers and teaching using both observation and student questionnaires (Wubbels and Brekelmans, 2005). Their questionnaire was subsequently adapted for use in higher education and indicated that students preferred teaching based on *emotional proximity –* directing, helping, supporting, and understanding (Fraser et al., 2010). In other research, the main characteristics of *outstanding university teachers,* as described by other academics, were “recognizing the student perspective,” “creating a learning ethos,” and “conveying feelings and arousing interest” (Ballantyne et al., 1997). This latter aspect also implies “authenticity” (Kreber, 2007); teachers openly express enthusiasm for the discipline and talk about their own ideas, values, and feelings about the subject, as well as showing a warm regard for students.

The phenomenographic approach to research (Marton and Booth, 1997) provides a way of systematically differentiating between different approaches to teaching through in-depth interviews with university teachers. Using this approach, Prosser and Trigwell (1999) identified five categories of approaches to teaching, which ranged from a predominant focus on *transmitting information* to *encouraging conceptual change*. In this nested hierarchy, lower levels are progressively incorporated within higher ones to offer increasingly more sophisticated conceptions of teaching. A sixth category subsequently added a category of “relationships between teachers’ world-views and students’ world-views, seen as open to change” (Prosser et al., 2007, p. 54; Prosser and Trigwell, 2014). This last category is closely linked to the ideas about teacher-learner relationships explored in the current study.

Cognitive and affective characteristics of university teachers were brought together within a “sophisticated conception” of teaching and learning in research by Entwistle and Walker (2002), based on differing forms of teacher knowledge identified by Shulman (1987). The cognitive elements were seen as “conceptualizing the topic and the discipline,” “strategically linking teaching with learning,” and “understanding how students learn,” while the affective characteristics involved “expressing feelings for the discipline,” “commitment to fostering conceptual development,” and “showing empathy with students.”

Research specifically focused on students’ *perceptions of teaching* has been widely reported in the literature (e.g., Ramsden, 1979; Prosser and Trigwell, 1999) and has demonstrated a clear influence of such perceptions on students’ approaches to learning and grades awarded.

### Student Learning

The range of research into student learning involves a wide range of perspectives, but here we focus mainly on the strand of this research, stemming from the work of Marton and his colleagues in Gothenburg (Marton, 1975; Marton and Säljö, 1984). They introduced the distinction between deep and surface *approaches to learning*. Initially, this idea was related to a specific experience of learning but was subsequently extended to fairly consistent approaches found across similar learning tasks. These approaches are related to high achievement at university, at least when “understanding” is central to assessments. Central to the notion of approaches is the *intention* of the learner when deciding which learning processes to adopt, and the *level of understanding* being sought. Interview studies with university students produced a nested hierarchy of six levels: *mentioning, describing, relating, explaining, conceiving, and expansive awareness* (Entwistle, 2018). The final category involves a broader understanding of the discipline as a whole and a readiness to reinterpret it within new contexts and to establish a personal relationship with the phenomena being understood (see also, Fyrenious et al., 2007; the final category described by Van Rossum and Hamer, 2010).

Earlier, Perry (1970) had described students’ experiences of learning by tracking their evolving conceptions of knowledge during a whole degree course. He introduced the distinction between *dualism* and *relativism,* which evolved gradually as students recognized the importance of justifying their conclusions with evidence and logical reasoning.

Recent research has looked at a range of individual factors (Gijbels et al., 2014; Postareff et al., 2014; Postareff et al., 2015) along with the strong feelings that students express about their *aspirations* and *sense of identity as a learner* (Haggis, 2007; Karagiannopoulou, 2010), with other studies concentrating on emotions expressed by students in their learning experiences, with “enjoyment” and “relief,” for example, contrasting with “boredom” and “anxiety,” and all of these feelings being related to the level of academic performance (Pekrun et al., 2011; Trigwell et al., 2012; D’Mello et al., 2014; Postareff et al., 2017).

Hagenauer and Volet (2014) have recently suggested that future research should focus directly on conceptualizing the influence of student-teacher relationships on students” learning (Rowe et al., 2013; Raisanen et al., 2016). An exploratory study along these lines identified a *meeting of minds,* as a particular form of learning relationship between teachers and their students (Karagiannopoulou and Entwistle, 2013), and suggested the need for further such research.

## Theoretical Framework

**Figure 1:**
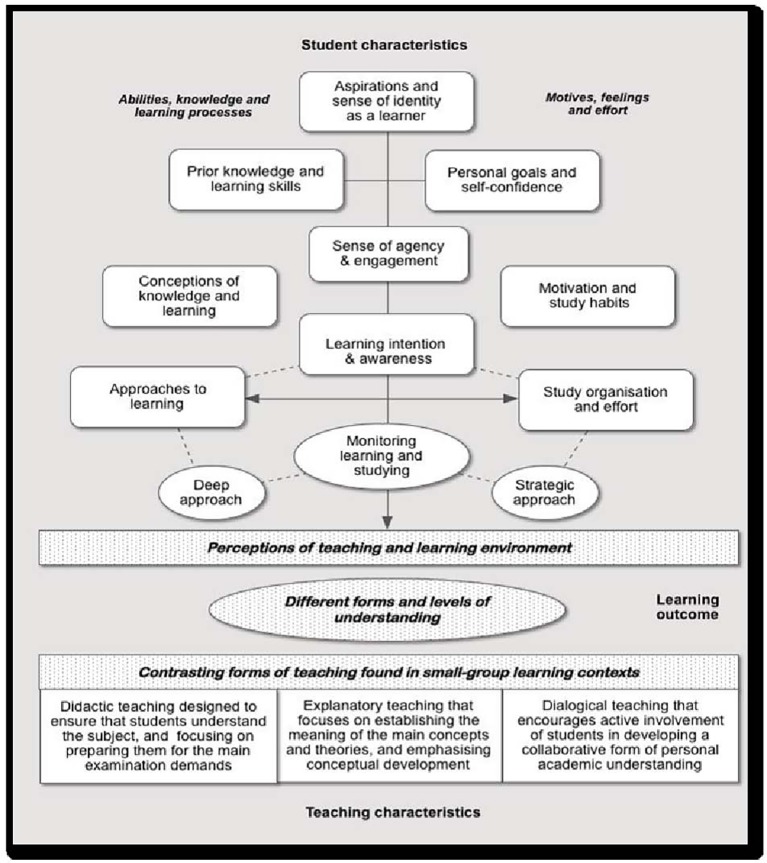
Model of student and teaching characteristics and learning outcomes.

Empirical research into student learning, derived from Marton’s ideas, led to a series of theoretical frameworks showing the influences of student characteristics and teaching approaches on learning. Biggs (1987) (see also Biggs and Tang, 2007) introduced a “presage-process-product model” (developed by Prosser and Trigwell, 1999) indicating a flow of influences on the quality of student learning. Figure 1 is developed from other more detailed conceptual frameworks based on similar principles (Entwistle, 1987; Entwistle and Peterson, 2004).

The vertical line in the top half of the diagram follows a time sequence beginning with students’ aspirations and sense of identity as a learner, through a sense of agency in learning, to specific learning intentions and awareness of the learning process, through which the monitoring of learning takes place. These processes are seen to affect students’ perceptions of teaching, which in turn relate to the different forms and levels of understanding reached. The terms *didactic* and *explanatory* come from the literature described above, but the idea of *dialogical* comes from, among others, Hay (2010).

## The Present Study

The present study explores the relationships between differences in student learning characteristics and perceptions of contrasting approaches to small-group teaching. It was based on previous research, but particularly on the findings of our previous exploratory study (Karagiannopoulou and Entwistle, 2013), which had introduced the notion of a *meeting of minds* between student and tutor and suggested that there might be striking differences among the students in their perceptions of small-group teaching, as illustrated in the following interview extracts from that study.

In the past I presented my own perspective in the exam, I was critical to the theories and I failed. Now, I develop my answer close to the tutors’ ideas, adding only few personal thoughts, if necessary. They treat us like machines; we’re asked to regurgitate knowledge. I’m always seeking meaning, but I can’t be bothered any more to develop my understanding in ways that meet the tutors’ demands.

*I try to take a critical stance on the material. The germ of it can be found in tutor’s thinking, which is ‘feeding’ mine. This gets me into more thinking. I initially try to understand the issue, by putting myself in the tutor’s shoes, how she appeared to think on an issue. You start with the tutor’s perspective, you bring in previous knowledge and experiences, and that gets you to a different end point from where you started*. (Karagiannopoulou and Entwistle, 2013, pp. 90, 91)

Both these students had a strong intention to reach a personal understanding of academic material but had experienced contrasting forms of teaching. The present study was designed to explore this finding in more depth, and with a larger sample, strategically chosen from highly successful students, more likely to have experienced a “meeting of minds” with tutors.

## Methodology

### Research Questions

1. How do students with differing learning characteristics perceive contrasting approaches to teaching?
2. What are the perceived characteristics of teaching that specifically encourage a “meeting of minds”?

### Sample and Educational Context

Twenty-one final semester Honor students (18 females and 3 males) were selected, all of who had top grades and were taking a joint degree in Philosophy, Education, and Psychology. In Greece, the gender ratio in Schools of Social Sciences is overwhelmingly in favor of women. All of the students had taken psychology as a major. The mean age of the participants was 21.5 years (*SD* = 0.4). All of the students were studying their first degree; no student having completed a previous degree participated in the study. The Honor students were identified through the University records. In final-year teaching in this department, students are taught in small groups, where they present papers, take part in thematic groups, and are trained in specific skills. This context enables a close relationship to develop between students and tutor, although not all tutors necessarily adopt that approach.

### Data Collection

The interviews were carried out by one of the authors who was a university teacher. However, the participants had not taken any of the courses she had been teaching in the year the study was conducted. Moreover, they were not expected to enroll in new courses until the completion of their studies; they scheduled to get their degree in the next months. The interviewer is an experienced researcher who has carried out and published a number of interview studies (e.g., Karagiannopoulou, 2006; Karagiannopoulou, 2010; Karagiannopoulou and Entwistle, 2013; Entwistle et al., 2016). Individual semi-structured interviews, lasting up to 90 min, used the conversational style adopted in phenomenographic studies, in which students are encouraged to reflect on their own experiences in depth and to clarify their initial descriptions. The interview schedule was designed to obtain comparable data from all respondents and covered, first, their learning characteristics and then experiences of different kinds of teaching, although both aspects were often interwoven in the student’s experience. In particular, the interview schedule included few core questions about students’ individual characteristics – elements of their learning identity, a variety of teaching experiences either interwoven or not with learning experiences, and the relevant emotions. Given the dialogic style of interviewing, almost any single specific question was followed by a number of probing questions that enabled the interviewer to gain deep understanding of students’ experiences; negotiate, with the students, to produce a shared meaning; and facilitate any attempt for meaning making. The questions involved the small-group psychology classes the students had attended so far at degree level. These were optional psychology classes. Students reported experiences of teaching and learning in relation to different tutors who delivered these small-group classes. No data were collected from the tutors. It should be pointed out that ethics approval was not required at the time this study was conducted as per the applicable institutional and national guidelines and regulations. Written informed consent was obtained from all participants.

### Data Analysis

Unlike the phenomenographic analysis of interviews, the transcripts were considered in relation to the pre-existing constructs shown in the theoretical framework. Repeated reading of the transcripts identified extracts that contained the essence of the meaning of the main constructs, related to both learning and teaching, that were then related to each other to explore the inter-relationships between them.

### Findings

The interviews had shown that students could recognize the three approaches to teaching shown in Figure 1 and describe their reactions to them. The findings are briefly summarized in Table 1 to provide an initial indication of responses found in the analyses.

**Table 1:** Outline findings indicating the perceptions of teaching of highly successful final-year students, differing in their learning intentions.

Students’ learning intentions and conceptions of knowledge	Group 1 (*N* = 10)	Group 2 (*N* = 7)	Group 3 (*N* = 4)
Perceptions of teaching	Deep learners seeking a wider and deeper academic understanding, with a strong relativistic conception of knowledge	Deep learners seeking an individual understanding of concepts and theories, with a clear relativistic conception of knowledge	Less enthusiastic learners content with meeting assessment requirements and showing little recognition of relativism
A. Didactic teaching to provide sufficient concepts and theories to cover the syllabus	Dissatisfaction, but tolerance	Dissatisfaction, insecurity, and frustration	Satisfaction
B. Explanatory teaching to encourage and support students’ understanding	Satisfaction	Satisfaction	Satisfaction
C. Dialogical teaching to offer a “meeting of minds” and share the exploration of understandings	Excitement	Satisfaction	Confusion and insecurity

We found three distinguishable groups of students in terms of learning characteristics, mainly on the basis of approaches to learning, level of intended understanding, and conceptions of knowledge but also on evidence of a learning identity. The perceptions of teaching were related to the three distinctive approaches. The relationships between student characteristics and perceptions of teaching were then examined within the three groups of students making use of the whole transcript for each student. Summaries of these analyses are provided in the following sections to justify the three student groups and their tendencies toward differing reactions to the contrasting approaches to teaching. Each section is illustrated by short extracts from the transcripts.

## Characteristics of the Three Groups of Students Identified

### Group 1 Deep Learners Seeking a Wider and Deeper Academic Understanding

All of the students in GROUP 1 reported a clear learner identity as active learners and were meaning oriented, seeking to reach a broad, independent understanding of the world around them, which could then be used to address important issues affecting people and the world. They also showed a strong relativistic conception of knowledge.

**M:** Learning is about relating theory to the real world and enhancing self-awareness. Critical reflection and comparison of the main ideas between different perspectives get me gradually to the development of a particular personal perspective, which is always under discussion. [I’m trying] to reach self-awareness, to challenge even my ground rules, de- and re- construct them through the lens of new perspectives. What matters for me are the academic experiences that ‘lead’ me to act as a citizen, thinking reflectively and challenging ideas. Lectures are what I call ‘food for thinking’: something to start from.

### Group 2 Deep Learners Seeking an Individual Understanding of Concepts and Theories

Students in GROUP 2 appear more pragmatic deep learners, as the aim to develop personal understanding seems to exist alongside a concern about being successful in the exams. They do, however, seem to have a clear awareness of the relativity of knowledge

**P:** Personal understanding is bound to be my main concern in order to get an ‘A’, [but] I’m the sort of student who wants to have the whole picture of an issue, that’s mainly the reason I don’t miss any lecture, [as] this is of so much help in the exam [revision].

**Z:** Academic work is about thinking about the other’s perspective and reflecting on the information I come across. I’m likely to express my own ideas in ways that other people have not been thinking about, and possibly shift their thinking to a different direction. [But] I need the lectures to show me the way to it, motivate me to do some work on it to get there.

### Group 3 Less Enthusiastic Learners Content With Meeting Assessment Requirements

Students in GROUP 3 studied in order to understand the main concepts. They lacked the confidence to try to reach a personal understanding, worrying that being “off track” would lead to failure in the exams. There was little evidence of a relativistic conception of knowledge.

**V:** I need to understand the concepts [I’m meeting] and make some links to previous knowledge. I doubt I can go further. I’m the sort of student who attends the classes expecting the tutors to stimulate her interest, get her deeper into the subject and do well in the exams. I may or may not be able to see things from the tutor’s perspective, but I’m always keeping in mind what I [have to] present in the exams.

## Perceptions of Approaches to Teaching and Influences on Learning

These analyses were directed toward the initial research question: “How do students with differing learning characteristics perceive contrasting approaches to teaching?” The perceptions that each of three groups of students with contrasting learning characteristics had the three perceived ways of teaching, identified in Figure 1 and Table 1.

1. Didactic teaching designed to provide sufficient concepts and theories to cover the syllabus and preparing the students for the exam.
2. Explanatory teaching that focuses on establishing the meaning of the main concepts and theories and emphasizing conceptual development.
3. Dialogical teaching to offer a “meeting of minds” and freedom to explore academic understandings, both collaboratively and individually.

The first two categories correspond to the last two categories identified by Prosser et al. (2007), mentioned earlier, but our previous study had suggested a broadening of the most advanced category, acknowledging the importance of learning relationships in approaches to teaching, to open up the possibility of a “meeting of minds.”

### Didactic Teaching

All three categories of teaching were perceived as being *student-focused* (Prosser and Trigwell, 1999) to varying degrees. In *didactic teaching*, however, the teacher was found to be taking more responsibility for the student’s learning than in the other two categories.

#### Group 1 Deep Learners Seeking a Wider and Deeper Academic Understanding

This group appeared immune to what they perceived as rather negative teaching experiences. They reported dissatisfaction, but also tolerance, in their reactions to this approach to teaching. They explained how their strong commitment to the development of personal understanding allowed them to engage actively with the ideas encountered without being unduly influenced by the teaching method. This was markedly different to how students in the other groups described their reactions.

**I:** My work on a subject cannot depend on the teaching style [experienced]. I may hate the style, but I’m determined to work on a particular issue in which I’m interested. I can’t avoid speaking my mind and having the opportunity to contribute to class discussions in a class where the tutor is not only presenting the main concepts but discussing perspectives: [it] tidies up my mind, sorts out misunderstandings. Such an experience enhances my confidence concerning the quality of my understanding and my potential to think in an academically appropriate way. It’s all about thinking in relation to the other’s perspective, which I miss when the tutor delivers only the main concepts and the core subject information.

#### Group 2 Deep Learners Seeking an Individual Understanding of Concepts and Theories

Students in this group appeared to be generally dissatisfied by this teaching approach, with some regression in their learning approaches. They were disappointed by the rather closed approach to the subject with students not being allowed to challenge the ideas presented. The teaching also failed to stimulate their interest or get them engaged with learning, sufficiently to come to grips with the subject. There was no evidence of a learning relationship between teacher and student that could encourage independent learning.

**E:** [With this sort of teaching] I can’t see things from a different perspective; it seems to be only one way to see a theory. I need extra information or examples to enable me to find something I’m interested in, so that I can keep working towards ‘real’ understanding. I don’t get into thinking in relation to her perspective and I don’t question what is presented in the class, since they present the main concepts as ‘golden’ ground rules of the discipline that can’t be challenged. So I miss the opportunity to get into more depth and feel confident with the development of personal understanding: I can’t risk going far with personal understanding, as I may be off track. I get disappointed and feel devalued, and may stop attending the lectures or [fail to] concentrate.

#### Group 3 Less Enthusiastic Learners Content With Meeting Assessment Requirements

All the students in this group were satisfied with tutors who explained the content sufficiently well for their learning intentions, with their focus being simply to succeed in the course. These experiences appeared related to a continuing dualistic conception of knowledge, with little expectation of becoming involved with the subject matter independently.

**S:** It’s not necessary to find something that makes sense to you in the lecture and say, “Yes, that’s me, I’ve seen this happening,” and become enthusiastic. Just sitting there and listening to the lecture is not bad at all. If you go there, you attend the lecture and you make sense of the content, that’s it! I need teachers to present some links with previous knowledge and give me some examples. It’s not the relationship [with the tutor] that facilitates my understanding, but the valid knowledge I get from the lecture that facilitates my study and examination preparation.

### Explanatory Teaching

This category is similar to the highest one described by Prosser and Trigwell (1999). Teachers tend to see learning from the student’s perspective and encourage personal understandings of the subject matter, but without forming a strong learning relationship with the students.

#### Group 1 Deep Learners Seeking a Wider and Deeper Academic Understanding

These students reported satisfaction with tutors who valued their own patterns of thinking. They tried to get close to such tutors in presenting their thoughts and get in touch with their own personal way of approaching the discipline that enabled them to experience a meeting of minds to which students themselves felt they had a seminal contribution. They felt valued and respected when the particular teaching experiences met the students’ need to demonstrate their own understanding and share thinking patterns, and yet missed a sense of “passion” in the teaching.

**C:** From my perspective, there’s nothing wrong with teaching as long as the meaning comes down to the students and there is a climate that allows students to express their thoughts and ask questions to clarify meaning. But the most important thing, that gets me further with meaning and exploring more aspects of the subject, [goes beyond this] to share with tutors their experience of how disciplinary principles become intertwined within their everyday life and their personality [and with] a passion that goes beyond a focus on students’ understanding. I’m so determined to get into grips with meanings that I always try to get really close to the tutor and share her perspective, while also expressing my thoughts and interests. Making sure that my way of thinking meets her very personal ways of dealing with the discipline is the “break through” point [for me]!

#### Group 2 Deep Learners Seeking an Individual Understanding of Concepts and Theories

These students appeared satisfied by the teaching delivered, as it enabled them to reach an independent form of understanding. The tutor’s concern about their learning, and readiness to be available, supported their ability to reach understandings of their own.

**Z:** It is not how many different perspectives she presents or how passionate she is with her discipline that makes me happy with teaching and encourages me to get involved with meaning making. [Rather it] is whether she is really concerned about her students following the thread of her thinking in order to develop an understanding, and whether she is open to questions and explains and discusses in depth the issues we raise. [It is also] whether she makes herself available, so I feel free to present my thoughts and get some feedback.

#### Group 3 Less Enthusiastic Learners Content With Meeting Assessment Requirements

These students reported satisfaction with teaching for understanding by taking the tutor’s perspective as an understanding to hold on to. They respected tutor’s personality and knowledge, creating a positive emotional experience supporting the students’ faltering steps toward understanding of the tutor’s perspective, but they lacked the confidence to interact with tutors and aimed at reaching a level of understanding sufficient to succeed in the exams.

**V:** I’m really satisfied with tutors who teach for students’ understanding, facilitating links with previous knowledge. This is important for me because I want to understand the lectures and leave the class with a feeling of satisfaction about my understanding. I see her as a model to identify with. As a consequence, I identify with her perspective, even though I may disagree with it. I’m not the type of student who asks questions; I’m not confident enough to do that, but I benefit from other students’ questions and answers. I like listening to the class discussion and overlooking the break-time discussions. Such experiences make me think that something important happens there, and I may reflect on it later on.

### Dialogical Teaching

This category not only contains elements of the sixth approach to teaching identified by Prosser et al. (2007) but also expands its defining features substantially, by introducing the idea of a “meeting of minds” in the learning relationship. The analysis of this category was directed toward our second research question: “What are the perceived characteristics of teaching that specifically encourage a ‘meeting of minds’?” The clearest evidence came from students who were seeking the most advanced forms of personal understanding (Group 1) and is described in detail in the next section.

#### Group 1 Deep Learners Seeking a Wider and Deeper Academic Understanding

All of the students in this group reported themselves to be excited by enthusiastic, empathetic teaching that appeared significantly to promote the relational nature involved in developing “expanding awareness” as part of their understanding. The main additional aspects of this approach to teaching were found to involve *tolerance of ambiguity, authenticity, empathy, being available, and creating thinking space,* which will now be described in turn. These students also provided some of the best explanations of how, specifically, the supportive context provided, and the interaction with the teacher’s ideas, enabled them to reconsider their previous understandings and build something more personally satisfying.

## Tolerance of Ambiguity

This characteristic can be seen in a tutor’s *receptivity* to any pattern of thinking presented by a student in response to a specific issue. Such an experience seems to validate students’ own understanding, as it comes from an expert who herself appears to be struggling with ideas and different perspectives within the disciplinary norms. Students perceived teachers’ response as eagerness to engage with their perspective – it involves mutuality: students felt respected and respectful.

**Ch:** [I appreciate] interaction with a tutor who responds eagerly to the ideas and thoughts I bring up. Supporting connections to an issue makes me feel that I’m on track and meaning can be reached, although I can hardly see it [initially]. Different ideas competing, even “irrelevant” ideas, can survive in this context: it’s all about confidence, feeling safe to struggle with meanings. I feel that my thinking stands up well, so it is worthwhile to keep thinking and struggling with meanings. My ideas matter to an expert - a valid person!

## Authenticity

Teachers sharing with students their own personal experiences of meaning making and their way of living appeared to present what students sensed to be their “*true self.*” Students experienced, vicariously, the ambiguity and uncertainty underlying the tutor’s personal process of becoming an expert in the discipline, which allowed them to feel confident when having similar feelings. The tutor’s eagerness to share personal experiences with students created feelings of trust, value, and reciprocity, which enhanced students’ confidence in struggling with disciplinary forms of understanding.

**Ch:** Teaching concerns her as a whole: it’s her personality that makes the difference, herself as a human being with particular interests, the true self she brings to the lecture in terms of how the disciplinary ideas have been involved in her life and how she lives with them, experiences them, explores them, sticks to them, faces difficulties, discusses with colleagues. … Sharing one’s personal experiences of dealing with knowledge brings the disciplinary concepts and difficult meanings down to the earth.

## Empathy

This characteristic could be seen in students’ reflections on the *shared meaning making*. A tutor’s passion for their discipline was perceived to go hand in hand with her concern and eagerness to get students contributing to the flow of meaning making that contributed to their intellectual development. They provided clear explanations of the multiple facets of an issue that enabled students to join in a classroom discussion during which the tutor was seen to be drawing from all aspects of the discipline know-how, justifying their own perspective and showing their love of the subject. Reciprocity in respect of emotion and cognitive activities was underlying such experiences.

**Ch:** She is pretty much passionate about the subject and its ideas. At the same time, she is concerned about me as a student, [about how I can] get the most out of [the subject], get into the heart of the discipline, [and] share my thinking patterns with her. That’s why she finds ways to integrate my perspective in the flow of meaning making and tries to get us all to participate in it… Her eagerness to get me into [the subject] makes me feel excited and confident to get further the meaning; it’s her eagerness to meet my thinking.

## Being Available and Providing “Thinking Space”

Students appreciated the time that tutors spent with them, particularly in break time when tutors provided opportunities for them to express their thoughts and to “appropriate” meanings within the disciplinary discourse. Such experiences appeared to validate their thinking and enhanced confidence. Tutors were perceived as supporting students to make the most of teaching in terms of their engagement with the disciplinary discourse and providing opportunities in class for students to contribute their own patterns of thinking for general discussion.

**Ch:** She is not teaching just for the sake of teaching. This is particularly apparent in break-time [when] she spends with students and during personal meetings. She makes time for us; such conversations clarify meanings because she is available to listen to us and we feel free to express any pattern of thinking that she then ‘positions’ within the disciplinary discourse. It’s like tidying up the mess in my mind.

**B:** She’s teaching in a way that creates space for the students to develop their own thinking. She invites us to challenge and reflect on theories; this enables me to think about all these and get into implications of it for my life.

These four aspects help to clarify the overall meaning of a “meeting of minds,” through which students shift toward an appropriate way of thinking, drawing on their engagement with the tutors’ perspective and their personal attributes. This process leads to a warm tutor-student learning relationship, which enhances students’ confidence to struggle with meanings. Clarification of explanations and negotiation of meanings appear to go hand in hand with feelings of concern and support. Experiencing such teaching allows students to think confidently in relation to the tutor’s perspective and so support the “expanding” form of understanding.

### Group 2 Deep Learners Seeking an Individual Understanding of Concepts and Theories

Like the first group, all these students reported satisfaction with enthusiastic teaching and pointed out how all aspects of students’ learning relations to tutors enhanced their confidence in developing personal understanding. However, in contrast to that group, these learning experiences did not help these students to share an already developed individual pattern of thinking or their steps toward understanding. They did, however, provide a starting point for thinking that kept them engaged with learning, helping to move the students’ understandings toward “expanding awareness.” Again, reciprocity in emotion and cognitive activities can be seen in the responses, implying an experience of a *meeting of minds* and feeling motivated by the tutor’s passion about her subject.

**P:** When the students present various ideas and perspectives [to the class] and the tutor is really happy to tidy them up and give meaning to them, [then] I start thinking in relation to [the tutor’s] perspective, and I may either adopt her perspective or challenge it. The more passion I experience from the tutors, the more interested I become in the issue at hand, and the more meanings I explore… We’re likely to get motivated by [the tutor’s] passion [about her subject].

### Group 3 Less Enthusiastic Learners Content With Meeting Assessment Requirements

Students in this group seemed to become confused and left behind by passionate teaching, feeling that the tutors were not taking the students’ pace of learning into account, but rather enjoying their own ways of thinking about the subject. Confusion was followed by negative feelings and reduced effort in learning, with no evidence of any experience of a meeting of minds.

**S:** The tutor is absorbed in the subject she is teaching and I feel like I’m left behind [in] meaning making. I don’t get upset, but eventually I pretend to attend without attending; it’s like passive aggression. I’m there sitting at the desk but I’m not concentrating on the lecture, so I’m destroying her lecture, in a way. I would prefer a down-to-earth tutor who is teaching in a way that I can understand; that’s my main concern as a student, to reach sufficient understanding to take with me when leaving the class, so that I may continue my work later on, [although] I may not.

## Discussion

This study addressed the two main aspects indicated by two research questions. The first asked whether there were differences among students in their perceptions of the three distinct approaches to teaching indicated in our theoretical model. The responses from students, with contrasting learning intentions and conceptions, indeed showed systematic differences across the teaching approaches. The second asked about the teaching characteristics that were seen to encourage a “meeting of minds.” Students, who were enthusiastic about “dialogical” methods of teaching, saw teachers using those approaches as showing, particularly, tolerance, authenticity, and empathy. Full descriptions of these findings are discussed below.

## Students’ Reactions to Different Approaches to Teaching

Table 1 summarized the main findings. Deep learners, who were seeking a wider and deeper academic understanding, were excited by *dialogical teaching* that encouraged a “meeting of minds.” They were, however, tolerant of the other forms of teaching, feeling they were well able to develop their own understanding with or without the ideas presented by the teachers. Deep learners looking for an independent understanding, but with a greater focus on exam requirements, were deeply dissatisfied by *didactic teaching* but satisfied by the “explanatory” and “dialogical” approaches. The dialogical teaching left the less enthusiastic learners who were focusing mainly on the exams, anxious and confused, but they were comfortable with both the other approaches to teaching.

One source of these differing reactions appears to lie in the very different learning identities (Haggis, 2007) of the students and their intentions and conceptions, as shown in Figure 1. Where there is *dissonance* between students’ learning intentions and a perceived approach to teaching (Lindblom-Ylänne, 2003), the tension creates negative feelings and confusion, as well as possible disengagement from attendance and studying (Coertjens et al., 2016; Postareff et al., 2017). In our own study, such dissonance was particularly strong among the less enthusiastic students when faced with teaching designed to promote a “meeting of minds.” This approach therefore has its dangers for students whose lower aspirations and knowledge may not allow any effective “meeting of minds” to take place. The dissatisfaction shown with “didactic” teaching by students aiming for their own personal understanding makes sense, as it is likely to discourage them from the ways of thinking they want to follow. This led to some of the strongest expressions of discontent, even anger, among this group of students.

Our findings also reinforce the importance of positive feelings of “consonance” (Haggis, 2007; Vermunt, 2007), where learning intentions and approach to teaching match each other. Among our students with the deepest learning intentions, teaching for a “meeting of minds” was mentioned enthusiastically, as they enjoyed interacting actively with the academic ideas of the tutor, as they developed their own understanding, testing those ideas out for themselves against their own previous knowledge and experiences. Such students also seemed to be immune from the potentially damaging effects of dissonance, through their confidence in their own ability to understand for themselves.

## Perceived Characteristics of Teaching that Encouraged a “Meeting of Minds”

In this study, we have been exploring the perceived characteristics of tutors who encourage a meeting of minds within the specific context of final-year, small-group, social science teaching. Such a context allows teachers to have more freedom in their teaching and to go beyond the level of basic academic understanding to enable some students to participate actively in formulating ideas within their discipline (Baxter Magolda, 1999; Kreber, 2007). Of course, the nature of the discipline will affect how possible it is for such sharing of ideas to take place.

The explanations provided by the students who were seeking a wider and deeper academic understanding provided the best source of evidence to describe the characteristics of teachers who encouraged a “meeting of minds.” The analyses established a range of aspects that could be categorized, although the specific extracts provided often overlapped with more than one category. The main categories described teachers who encouraged what we saw as a “meeting of minds,” as having *tolerance of ambiguity*, which involves openness to students’ ideas, *authenticity* in showing feelings and sharing experiences, and above all *empathy* showing a warm regard for students and expressing consideration for their views. But these teachers were also ready to be *available* during and after class to discuss academic topics, and students’ other concerns, and in class provided “thinking spaces” where students could reflect on and discuss new ideas that had been introduced.

Some of the students explained how the feelings expressed by the teacher affected them as learners. For example: *The more passion I experience from the tutors, the more interested I become in the issue at hand, and the more meanings I explore.* Teaching through a *meeting of minds* can be described as *relational teaching,* involving both mutuality and reciprocity, building bridges between the teachers, the students, and the subject. Students’ experience of “thinking in relation” involves the power of affect (care for students and passion for the subject), which can be utilized in encouraging “expanding awareness” in students’ understanding; experiences allow students more readily to make their own contribution to knowledge and to work within their professions.

Students’ perceptions of such characteristics correspond to many of academics had noted in outstanding teachers, namely “recognizing the student perspective,” “creating a learning ethos,” and “conveying feelings and arousing interest” (Ballantyne et al., 1997). Drawing on the work of Wubbels and Brekelmans (2005), Fraser et al. (2010) found that students, in general, preferred teaching based on *emotional proximity –* directing, helping, supporting, and understanding (Fraser et al., 2010). What our research has described is the different ways in which students with contrasting learning intentions react to teaching which has this range of characteristics. We have to be aware that these differing reactions imply that teaching has, as far as possible, to be congenial and helpful for students of differing levels of previous knowledge, ability, and intentions. This is difficult to achieve, but such teaching has been carried out, described as a *multipli-inclusive approach,* in which material or set-work of differing levels of sophistication is provided within the same course (Entwistle and Walker, 2002). In our study, some students’ difficulty to benefit from dialogic teaching seems to involve their individual characteristics; the difficulty can be seen to get overcome by a student-teacher relationship that allows an experience of a meeting of minds (emotional-cognitive teaching experiences) to take place. The four elements underlying the student-tutor relationship in the context of dialogic teaching, suggested in the present study, can be seen to allow emotional proximity that comes along with a multi-inclusive approach; students get involved in different levels of sophistication after having safely repeatedly undergone shared experiences of developing understandings.

The teaching experience involving a “meeting of minds” sheds light on Kreber’s (2007) suggestion for the need to provide students with a learning experience that is worthwhile and promotes their learning and development and a dialogue that centers on “ideas that matter.” It also supports previous studies indicating that friendliness and freedom enhance learning outcomes (Wubbels and Brekelmans, 2005; Fraser et al., 2010). Moreover, this teaching approach helps us to appreciate how students became better able to “shift” toward a disciplinarily appropriate way of thinking having safely “internalized” shared experiences of developing understandings. Students seem to move from mutual regulation in meaning making to a safe independent exploration of understandings, with the consequent development of “expanding awareness” in understanding. These aspects help us to make sense of previous findings that “thinking in relation” enables students to come to terms with alternative thinking paths in manageable “doses,” involving the exploration of both similarities and differences within a learning relationship where students feel valued and respected (Karagiannopoulou and Entwistle, 2013).

A caution, however, comes from the research of Wubbels and other researchers (Wubbels and Brekelmans, 2005; Fraser et al., 2010), which described personality characteristics that are favorably or unfavorably perceived by students. To the extent that such personality characteristics are stable, this suggests that teachers may well differ markedly in the extent to which they are ready, for example, to accept the sharing of ideas with students. However, teaching necessarily involves adopting a professional role, and also being adaptable, so this may not be a complicating factor.

Besides, it should be pointed out that the explanatory teaching seems to promote learning and be beneficial for any group of students. However, the dialogic teaching is suggested as one of the higher quality to the extent it promotes expansive awareness (Entwistle, 2018). The exploration of understanding that tutors share with students has been at the forefront of the development of personal understanding in higher education; it leads to a broader understanding of the discipline as a whole and a readiness to reinterpret it within new contexts and to establish a personal relationship with the phenomena being understood.

## “Meeting of Minds” As a Category of Teaching Approach?

Earlier, we raised the possibility that the idea of a “meeting of minds” might represent an additional category for the range of teaching approaches described by Prosser et al. (2007) and Prosser and Trigwell (2014), but it would be presumptuous to such a new category on the basis of a small-scale study in a single institution. Rather, we feel that it adds to the sixth category, mentioned earlier, by extending its defining characteristics which described such teaching as developing relationships between teachers’ and students’ world views and seeing them as open to change (Prosser et al., 2007. The sense of *openness* in teaching to allow ideas to be discussed in mutual interplay between teachers and students seems to be a good way of characterizing this sixth category of approach.

## Conclusions

The present study suggested three main perceptions of different types of teaching for students with different learning identities: (1) Didactic: explaining concepts and providing sufficient concepts and theories to cover the syllabus, (2) Explanatory: encouraging and supporting students’ understanding, and (3) Dialogic: providing a “meeting of minds” and freedom to explore understandings. The first two follow the set of categories of approaches to teaching presented in the relevant literature and are further developed to an enriched version of the sixth approach to teaching (Prosser et al., 2007) taking account of experiences of “meeting of minds.” This third category suggests a students’ experience of “thinking in relation” that promotes understanding in the context of emotional-cognitive teaching experiences. Students describe teachers in this category as having special qualities – *tolerating ambiguity, showing authenticity and empathy, providing opportunities for discussion* in breaks*, and offering thinking spaces* within class through which students feel free to follow their own lines of enquiry within the expected disciplinary discourse.

### Caveats

The main caveats involve the design and context of the study. It deliberately chose a group of successful final year students in order to focus on the nature of the “meeting of minds” found in the previous exploratory study, so it is impossible to generalize to a wider range of achievement. Moreover, the students were being taught in relatively small groups within which tutors had the opportunity to get to know the students individually, and they were being taught in a subject area – social science – in which personal experience could be drawn on and feelings were more openly involved. The teaching contexts in other subject areas have yet to be explored. Although the small sample and the qualitative data can be seen as important limitations of the study, the qualitative findings of the present study could inform a short subscale of approaches to teaching in order to provide quantitative data, which could confirm the present findings and allow the exploration of associations between “expansive awareness” and teaching in terms of a “meeting of minds.”

### Implications and Suggestions for Future Research

The findings of the present study seem to be in line with authenticity in scholarship of learning and teaching. Professional training that prioritizes the vital connection between teachers, students, and the subject could be benefited by the idea of “meeting of minds” as a teaching practice. The study could inform recent trends of scholars of teaching and learning in terms of “what matters” and students’ authentic motivation. The failure of an effective “meeting of minds” to take place in the third group demands a sophisticated conception of university teaching and learning, which may come only after considerable reflective experience of university teaching. The idea of a *multipli-inclusive approach,* which caters for students with a range of learning intentions and interests, by providing material or set-work of differing levels of sophistication within the same course, would seem to suggest another line of research to follow.

Future research might focus on the exploration of aspects of relational learning suggested by the present study. All these are in line with the development of bridges between teachers, students, and the subject. It would be important to discover the extent to which is found in other subject areas and what form it might take in those disciplines. Moreover, the primacy of emotions and the quality of student-teacher relationships underlying students’ learning should be further explored in the context of a “meeting of minds” in order to shed light on other subject areas and groups of students. The findings described here suggest that the idea of a *meeting of minds* represents a new construct that is open to further theorizing and conceptualization, leading toward the development of more powerful explanations of the influences of teaching on learning at university (Hagenauer and Volet, 2014), the development of “expansive awareness” (Entwistle, 2018). Moreover, it seems to be in line with the “open” discussion on the scholarship of teaching and learning (Kreber, 2007).

## Author Contributions

EK contributed to the theoretical framework of the article and carried out and analyzed the interviews. Also, she developed the first draft of the discussion. NE developed the full version of the introduction and the heuristic model and contributed a great deal to the summary table and the final analysis of the interviews. Also, he “refraimed,” to some extent, the final version of the discussion.

### Conflict of Interest Statement

The authors declare that the research was conducted in the absence of any commercial or financial relationships that could be construed as a potential conflict of interest.

The reviewer GP declared a shared affiliation, though no other collaboration, with one of the authors EK to the handling Editor.
